# Complete genome sequence of the wine strain *Saccharomyces cerevisiae* I-25 isolated from Cabernet Sauvignon sediment

**DOI:** 10.1128/mra.01228-25

**Published:** 2025-12-19

**Authors:** Alexey V. Beletsky, Egor A. Vasyagin, Maxim Yu. Shalamitskiy, Elena V. Ivanova, Nikolai V. Ravin, Andrey V. Mardanov

**Affiliations:** 1 Institute of Bioengineering, Research Center of Biotechnology of the Russian Academy of Sciences 204333 , Moscow, Russia; 2 All-Russian National Research Institute of Viticulture and Winemaking “Magarach” of the National Research Centre “Kurchatov Institute,” Yalta, Russia; Rochester Institute of Technology, Rochester, New York, USA

**Keywords:** *Saccharomyces cerevisiae*, wine strain, complete genome

## Abstract

Wine yeast *Saccharomyces cerevisiae* strain I-25, isolated from yeast sediment formed after spontaneous fermentation of Cabernet Sauvignon grapes in the Krasnodar region, is used to produce regional red wines in Russia. To characterize its genetic properties and biotechnological potential, we obtained a complete genome sequence.

## ANNOUNCEMENT

The yeast *Saccharomyces cerevisiae* is extensively used in various biotechnological applications and as a model organism for fundamental research (1, 2). Its importance in the production of alcoholic beverages (wine, beer, and other) stems from its ability to ferment sugars into ethanol and other metabolites that define the unique organoleptic characteristics of the final product (3). Significant differences between strains in their capacity to synthesize various secondary metabolites generate considerable interest in the selection of specific starter cultures to produce wines with different organoleptic properties.

A promising direction in yeast breeding is the exploration of autochthonous microbial populations from specific terroirs to discover strains with unique properties. However, a rational application of such isolates requires deciphering their genetic information. The goal of this study was the whole-genome sequencing and analysis of *S. cerevisiae* strain I-25 from the “Magarach” collection (4). The strain was isolated from yeast sediment formed after spontaneous fermentation of Cabernet Sauvignon grapes in a vineyard in the Krasnodar region in 1939. Homogenized sediment samples were serially diluted and plated onto 2% agarized grape must. After incubation at 25°C for 3 days, randomly selected colonies were inoculated into liquid medium. The strain was stored by repeated sub-culturing with incubation at 25°C for 3 days and storage at 10°C for 9 months. Strain I-25 was recommended for the production of red wines (5).

The strain was grown in YPD medium (1% yeast extract, 2% peptone, and 2% dextrose) at 28°C for 2 days (6). Genomic DNA was extracted using DNeasy PowerSoil DNA Isolation Kit (Qiagen, Germany). For genome sequencing, we employed a hybrid sequencing approach, integrating the Illumina and Oxford Nanopore platforms. A library for Illumina sequencing was prepared using the NEBNext Ultra II DNA Library Prep Kit (New England Biolabs, USA). Illumina sequencing was performed on a MiSeq instrument (Illumina, USA), producing 2,042,692 of 300 nt reads (total 612,807,600 nt). Adapter sequences were removed using Cutadapt v.4.0 with default parameters (7); overlapping paired-end reads were merged using FLASH v.1.2.11; “-M 300” parameter was set (8); and low-quality read ends were trimmed using Sickle v.1.33 (https://github.com/najoshi/sickle) with a quality threshold of Q30. In addition, the genomic DNA was sequenced on a MinION (Oxford Nanopore Technologies, UK). A genomic library was prepared from non-sheared genomic DNA without subsequent size selection using the Ligation Sequencing gDNA kit (SQK-LSK109) and sequenced on FLO-MIN110 flow cells. Guppy v.5.0.7 (Oxford Nanopore Technologies) was used for base-calling and quality filtering, with parameters selected automatically based on the flow cell and kit. A total of 305,290 reads with a mean length of 6,251 and N50 of 8,367 nt were obtained (1,908,367,790 nt in total). MinION reads were *de novo* assembled into chromosomes using Flye v.2.9.5 (9) with –nano-hq parameter, and the consensus of the assembly was polished with Illumina reads using the POLCA tool of the MaSuRCA v.4.1.4 assembler (10). As a result, we assembled the complete genome of *S. cerevisiae* strain I-25, consisting of 16 chromosomes and the mitochondrial genome. Genome statistics are described in Table 1.

**TABLE 1:** Genome sequencing statistics^*a*^

Parameter	Value
Illumina sequencing	
Read pair number	2,042,692
Total bases (bp)	866,773,190
Nanopore sequencing	
Read number	305,290
Total bases (bp)	1,908,401,477
Genome assembly	
Total base (bp)	13,278,158
Chromosome (length [bp])	
I	211,678
II	799,297
III	317,892
IV	1,487,001
V	562,483
VI	235,318
VII	1,047,440
VIII	528,305
IX	372,084
X	722,024
XI	665,831
XII	2,630,927
XIII	911,418
XIV	764,518
XV	1,030,074
XVI	912,007
Mitochondrion (bp)	79,861
GC content (%)	38.83
Protein-coding genes	5,283
tRNA genes	300
Non-coding DNA (%)	38.42
Genome coverage	225×
Copies of rRNA gene cluster	About 181
Genome assembly assessment (%)	
Complete BUSCOs	99.2
Fragmented BUSCOs	0.0
Missing BUSCOs	0.8

^*a*^ BUSCO, benchmarking sets of universal single-copy orthologs.

GeneMark-EP v.4.71 (11) with hints from protein splice alignments was used to predict gene structures. Hints were predicted using ProtHint v.2.6.0 (11) pipeline with the fungi subset of OrthoDB as a reference database. BLAT v.34 (12) splice-aware alignments of *S. cerevisiae* strain S288C genes to the assembled chromosome were incorporated to Augustus v.3.4.0. workflow as hints to improve intron-exon predictions. “--genemodel=complete—noInFrameStop --noInFrameStop=true” parameters for Augustus v.3.3.3 were specified (13). Genome completeness was assessed using BUSCO v.5.7.1 (14) with the *Saccharomycetes* database.

Comparison of strain I-25 genome with 1,689 complete genomes of *S. cerevisiae* from the NCBI database (accessed 27 September 2025) showed that closest genome belongs to *S. cerevisiae* strain Phaff 40-119 (GCA_037126535.1), with an average nucleotide sequence identity of 99.87%, calculated by FastANI v.1.33 (15). Default parameters were used for all software unless otherwise specified.

The obtained complete genome of strain I-25 will serve as the basis for further comparative genomics studies of *S. cerevisiae* wine yeast strains.

## Data Availability

The raw reads have been deposited in the NCBI Sequence Read Archive under accession numbers SRR35580637 (MiSeq) and SRR35580636 (MinIon). Assembled complete chromosomes and mitochondrion sequences were deposited in GenBank under accession number JBRBCX000000000. The version described in this paper is the first version.
